# The Role of Hydroxychloroquine in Coronavirus Disease 2019. A Versatile Tool at the Service of Humanity

**DOI:** 10.3389/fmed.2020.00176

**Published:** 2020-04-24

**Authors:** Argyris Tzouvelekis, Theodoros Karampitsakos, Demosthenes Bouros

**Affiliations:** First Academic Department of Pneumonology, Hospital for Diseases of the Chest Sotiria, Medical School, National and Kapodistrian University of Athens, Athens, Greece

**Keywords:** COVID-19, hydroxychloroquine, dose, efficacy, side-effects, coronavirus

Hydroxychloroquine is an old anti-malarial drug that has shown also efficacy in Q-fever (*Coxiella Burnetti*) and Whipple disease (*Thropheryma Whipplei*). Hydroxychloroquine has also been effectively administered in patients with systemic lupus erythematosus (SLE), rheumatoid arthritis and sarcoidosis with skin manifestations and refractory hypercalciuria. Hydroxychloroquine acts through increase of lysosomal pH in antigen-presenting cells and as an inhibitor of autophagy (process of selective degradation/removal of damaged organelles from the cell through the autophagosome). Anti-viral properties were also attributed to a mechanism involving interference with glycosylation of angiotensin-converting enzyme- (ACE)-2, the cellular receptor of SARS-CoV (1, 2). It shows high tissue absorption with a terminal half-life of almost 40 days that is mainly attributed to high-tissue deposition and not reduced clearance. Its major side-effects are vomiting, headache, changes in vision i.e., retinopathies, muscle weakness and QT prolongation.

*In vitro* studies have shown strong anti-viral activity of chloroquine (1, 3, 4). Chloroquine blocked virus infection at low-micromolar concentration and showed high SI (EC50 = 1.13 μM; CC50 > 100 μM, SI > 88.50) (4). Besides its antiviral activity, immunomodulatory properties of chloroquine might have synergistically enhanced its antiviral effect *in vivo*. In an effort to investigate the impact of timing on its immunomodulatory effect, authors demonstrated that chloroquine was effective both at entry and post-entry stages of the novel coronavirus in Vero E6 cells (4). Timing of administration has been a matter of debate, as hydroxychloroquine's anti-TNF action might have a detrimental effect in the early phases of the disease. On the contrary, immunomodulatory properties could have a cardinal role for the prevention of cytokine storm (5). This phenomenon could explain discrepancies in previous reports. With regards to studies investigating dosage, a dose projection was made from *in-vitro* experiments to humans and reported that an initial- loading dose of hydroxychloroquine 400 mg twice a day followed by 200 mg twice a day for 4 days could be effective in humans. Authors reported that hydroxychloroquine was more effective than chloroquine (lower EC50-0.72 vs. 5.47μM) (1).

In China, a panel of experts recommended chloroquine phosphate, administered orally at a dose of 500 mg twice per day for 10 days for patients diagnosed as mild, moderate and severe cases of novel coronavirus pneumonia and without contraindications to chloroquine (6). Regarding hydroxychloroquine, the first non-randomized, single-center clinical trial of hydroxychloroquine in humans was recently published by the French group of Didier Raoult and reported that the optimal dose is 600 mg administered as 200 mg thrice per day for 6 days (7). Authors enrolled 42 patients−26 were given hydroxychloroquine 200 mg tid (overall 600 mg) and 16 were the control group. Six patients from the drug-arm were lost in follow-up and thus analysis included an overall of 36 patients. Patients were predominantly male, of middle age (mean age of 45 year-old) with a mean incubation time of 4 days. The study met its primary end-point which was virological clearance, meaning PCR negativity, which was achieved in 70% of patients compared to 12% in the control group (*p* = 0.001). Interestingly, its effects were enhanced by azithromycin which was co-administered in 6 patients to prevent bacterial co-infection. All of these patients (100%) exhibited virological clearance compared to 57% of patients in the single-drug group. Intriguingly, one patient who was still positive following 6 days of hydroxychloroquine administration of azithromycin resulted in negative PCR 3 days after. Notably, two patients receiving hydroxychloroquine were asymptomatic. The study was underpowered and included non-severe cases of COVID-19, meaning non-ventilated patients. These findings were corroborated by a second study of the same group enrolling 1,061 patients with COVID-19. Combination of hydroxychloroquine and azithromycin, started immediately after diagnosis, led to a mortality rate of 0.5% in elderly patients^1^.

Results are encouraging but should be treated with caution. Perhaps hydroxychloroquine could be effective in combination with other drugs (i.e., azithromycin or other immunomodulatory or anti-viral agents) in most moderate and severe cases. The^1^ concept of using hydroxychloroquine as a preventive strategy in the general population is baseless and potentially hazardous. Our approach in Greece is to administer hydroxychloroquine in all hospitalized patients including those intubated in ICU, in combination with azithromycin as well as anti-viral agents depending on disease severity.

Hydroxychloroquine represents a cheap, relatively safe and potentially effective therapeutic option for COVID-19 respiratory tract infections. Its synergistic effect with azithromycin, another potent immunomodulatory agent which has shown effectiveness in Zika and Ebola viruses, requires further investigation. Future large randomized placebo-controlled clinical trials are eagerly awaited to prove this concept. Avoidance of irrational use by asymptomatic or mild-symptomatic non-hospitalized non-confirmed COVID-19 cases should be aggressively pursued.

## Author Contributions

AT wrote the initial manuscript. TK and DB were involved in article modification and offered intellectual contribution for the latest data in the context of COVID-19. All authors offered significant intellectual contribution and approved the final form of the manuscript.

## Conflict of Interest

The authors declare that the research was conducted in the absence of any commercial or financial relationships that could be construed as a potential conflict of interest.

## Footnotes

^1^https://www.mediterranee-infection.com/wp-content/uploads/2020/04/Abstract_Raoult_EarlyTrtCovid19_09042020_vD1v.pdf?fbclid=IwAR2Bx-Fldm2fFsLg7L06BYUz-8QvwuJ-hAW9uWmwx1KdKzCU0YGHtSwXXWw
